# Effects of Nicotine and Tobacco-Related Products on the Feeding Behavior of the German Cockroach (Blattodea: Blattellidae)

**DOI:** 10.1093/jisesa/ieaa147

**Published:** 2021-03-04

**Authors:** Vincenzo Di Ilio, Michael A Birkett, John A Pickett

**Affiliations:** 1 Biointeractions and Crop Protection Department, Rothamsted Research, Harpenden, Hertfordshire, UK; 2 BBCA Onlus, Rome, Italy; 3 School of Chemistry, Cardiff University, Cardiff, Wales, UK

**Keywords:** *Blattella germanica*, nicotine, tobacco smoke particulate, feeding behavior

## Abstract

Animals use olfaction to detect developmentally significant volatile organic compounds (VOCs) in their local environment. As part of a wider study aiming to demonstrate that the olfactory responses of animals to VOCs can be modified through the creation of a drug-addicted status and association with a selected VOC, we investigated nicotine and tobacco smoke particulate (TSP) extract as possible addictive compounds for male German cockroaches, *Blattella germanica* (Linnaeus). In feeding experiments using an artificial food stimulus, food treated with TSP extract was preferred over untreated food. Surprisingly, nicotine, which was expected to be the most important addictive tobacco component, did not induce noticeable effects on cockroach behavior. Both TSP extract and nicotine were shown to be phagostimulants. Olfactometry assays that measured odor-mediated insect behavior demonstrated that male *B. germanica* did not choose TSP-extract-treated food even when attempts were made specifically to train them via this modality. These results support a hypothesis that *B. germanica* needs to consume TSP-containing food to show a clear preference for this stimulus and that gustatory mechanisms are involved due to compounds present in the TSP extract.

The addiction of animals to psychoactive substances leads to modification of motivation priorities, replacing innate unconditioned stimuli with induced artificial needs that can be exploited in a classical conditioning paradigm. In the original model of learning, animals learn to associate an original neutral stimulus, the so-called conditioned stimulus, with a biologically active unconditioned stimulus or reward (Pavlov 1927). The reward elicits an innate response that is an unlearned physiological reflex. In classical conditioning, the innate response represents the expectation of the reward that comprises an internal representation of the reward in the absence of reinforcement by the cues and events predicting such a reward (Tolman 1959, Gil et al. 2007). In such a system, the value of the reward associated with a stimulus is not an intrinsic property of the stimulus itself and animals can assign different values to the stimulus in relation to their previous experience (Schultz 2000). In this context, the olfactory system of animals can be exploited, through associative learning processes, for the detection of volatile organic compounds (VOCs) that are unconnected with the lifecycle of the animals themselves (Suckling and Sagar 2011, Schott et al. 2013).

Building on these theoretical assumptions, we attempted to demonstrate modification of the olfactory priorities of animals through creation of an addicted status, using the German cockroach, *Blattella germanica* (Linnaeus), as the model species. Kaun et al. (2012) found that fruit flies, *Drosophila melanogaster*, are a useful model to understand the mechanisms underlying addiction. However, *B. germanica* was chosen as the model insect in our study because of its evolutionary and physiological features and because it is theoretically possible to associate olfaction with addiction. Cockroaches live in association with human populations in chemically complex environments, are sensitive to a huge spectrum of VOCs (Dow 1986, Bell 1990) and are able to discriminate odors in complex mixtures (Sakura et al. 2002). Cockroaches also exhibit an extremely flexible behavior that is associated with their ecological success (Sakura and Mizunami 2001, Lent and Kwon 2004, Decker et al. 2007). In this context, our aim was to identify cues of addiction that could be used as an ‘artificial’ unconditioned stimulus to be used in the context of a Classical Conditioning Pavlov’s paradigm. Nicotine was chosen as the addictive compound because it is readily available relative to other drugs of human abuse and is recognized as a potent addictive compound among mammals (Di Matteo et al. 2007, Benowitz 2010). Together with tobacco smoke particulate (TSP), which is also addictive to mammals, both materials were assayed as possible addictive substances for male *B. germanica*.

## Materials and Methods

### Insects

Gravid female German cockroaches, *B. germanica*, were purchased from i2L Research Ltd (Cardiff, United Kingdom) and maintained under constant temperature and humidity (28 ± 2°C; RH 60 ± 5%) with a photoperiod of 12:12 (L:D) h. Insect husbandry was carried out in 12-liter airtight containers equipped with a hole in the lid covered with a light metal net. The rim of the container was painted with Fluon PTFE (Blades Biological Ltd, Cowden, United Kingdom) to prevent escape. Water and ground dog pellets were provided as food ad libidum together with cardboard strips as harborage. Upon emergence, first-instar nymphs were collected and transferred into 1.7-liter containers with water, food, and cardboard harborages and allowed to reach the adult stage. Seven-day-old adult males were used for experiments, with males measuring ca. 1.5 cm in length and 0.5 cm in width.

### Chemicals

Nicotine (99% TLC purity) was purchased from Sigma–Aldrich (Gillingham, United Kingdom). TSP extraction from exhausted cigarette filters (*n* = 20) was carried out by soaking filters in ethanol (100 ml) at ambient temperature for 5 min. The extraction procedure was repeated three times, and the combined ethanolic extracts evaporated in vacuo to yield a dark brown residue (1.39 g). Solutions of nicotine in ethanol (50 mg/ml) and the TSP extract in ethanol (10 ml) were prepared prior to administration in artificial food.

### Nicotine Quantification

The amount of nicotine in the TSP extract obtained as described above was quantified by extraction of an aliquot with diethyl ether (three times with a double volume of diethyl ether) and analysis on an Agilent 6890 GC (Agilent Technologies, United Kingdom) equipped with a cool on-column injector, a flame ionization detector, and a nonpolar HP-1 bonded-phase fused silica capillary column (50 × 0.32 mm i.d., film thickness 0.52 µm). The oven temperature was maintained at 30°C for 1 min, programmed at 5°C min^−1^ to 150°C and held for 0.1 min, then increased at 10°C min^−1^ to a final hold at 230°C for 50 min. Hydrogen was the carrier gas. Results were obtained with an enhanced integrator (HP Chemstation). The amount of nicotine in the TSP extract was determined using an external standard method, i.e., by comparing the GC peak area with an external calibration curve prepared using different concentrations of nicotine.

### Nicotine and TSP Extract Addition to Cockroach Diets

Nicotine and TSP extract were administered to adult male German cockroaches in feeding bioassays by mixing with freshly prepared artificial food. Diets used in cockroach bioassays comprised 1) a liquid mixture obtained by mixing tap water (60%), yeast extract (32.5%), sucrose (6.5%), corn oil (0.2%), and 0.1 mg/ml potassium sorbate (0.8%) or 2) a solid food comprising the liquid mixture described above plus 1% agar. For the liquid mixture, nicotine or TSP ethanolic solutions (see above) were added at ambient temperature, such that the concentration of nicotine (0.5 mg/ml) was consistent between the liquid mixture and solid food. For the solid food, nicotine and TSP ethanolic solutions were integrated when the liquid mixture containing 1% agar reached a temperature of 50°C. Control food (containing just the liquid or solid diet and no nicotine or TSP extract), nicotine-treated and TSP-extract-treated food were prepared such that they each contained the same amount of ethanol when food was offered to insects.

### Bioassays

Bioassays with male German cockroaches, *B. germanica*, were carried out in controlled environment conditions under constant temperature and humidity (28 ± 2°C; RH 60 ± 5%) with a photoperiod of 12:12 (L:D) h. Three experiments were conducted:

### Experiment 1—Nicotine and TSP Innate Preference

The aim of this experiment was to define the innate preference of male German cockroaches for nicotine or TSP extract when administered with artificial food. The experiment was carried out in two distinct phases and is summarized in Table 1.

**Table 1.** Structure of experiment 1 showing the food regimes in the first phase and in the choice phase

First phase (3 d)	Choice phase (5 d)		
	Choice	Males/Rep.	Rep.
Starved	BLA vs NIC	4	4
	BLA vs TSP	4	4
Blank	BLA vs NIC	4	4
	BLA vs TSP	4	4

In the choice phase, male German cockroaches, *Blattella germanica*, were able to make a binary choice of the food source. BLA, artificial control food; NIC, nicotine-treated food; TSP, tobacco smoke particulate extract-treated food.

#### Phase 1

Two groups of eight males were randomly selected from the adult *B. germanica* colony 7 d after the last molt, with each group being placed each in a 1-liter airtight container equipped with a drilled lid for ventilation. Each container was provided with a water dispenser and a cardboard strip as harborage. The first group was starved for 72 h, whereas the second group fed on artificial control food (0.5 g) offered in solid form inside a 1.5-ml Eppendorf tube (Table 1). Food consumption was determined by weighing the tubes with feed before and after the experimental period. Water evaporation was also considered.

#### Phase 2—Choice Phase

In the second phase (Table 2), male cockroaches were placed individually into a round 1-liter airtight container and allowed to make a choice between two test solutions in liquid form administered by means of two 25-µl glass capillaries inserted through the container wall, according to a modified version of the two-choice capillary feeder assay (Ja et al. 2007). Cockroaches were allowed to imbibe the fluid from the exposed tip of the capillaries and the amount of food consumed was recorded daily. Briefly, the food consumed was calculated by subtracting the amount of food remaining in the microcapillaries from the original volume of 25 µl. Water evaporation was also considered.

**Table 2.** Structure of experiment 2 showing the food regimes in the first phase of training, in the second phase of stabilization, and in the choice phase

Training phase (7 d)	Stabilization phase (3 d)	Choice phase (5 d)		
		Choice	Males/Rep	Rep.
Tobacco smoke particulate matter (TSP)	Starved	BLA vs TSP	4	4
	TSP	BLA vs TSP	4	4
	Control	BLA vs TSP	4	4
Nicotine (NIC)	Starved	BLA vs NIC	4	4
	NIC	BLA vs NIC	4	4
	Control	BLA vs NIC	4	4

In the choice phase, male German cockroaches, *Blattella germanica*, were able to make a binary choice of the food source. BLA, artificial control food; NIC, nicotine-treated food; TSP, tobacco smoke particulate extract-treated food.

As shown in Table 1, both the starved and the fed adults could make only a binary choice between treated and control food. Therefore, starved males were divided into two subgroups: the first subgroup could choose between control food (BLA) and nicotine-treated food (NIC), whereas the second subgroup could choose between control food (BLA) and TSP-extract-treated food (TSP). Similarly, fed adults were divided into two subgroups, where choices were between BLA and NIC in the first subgroup and between BLA and TSP in the second subgroup. The experiment was repeated four times.

### Experiment 2—TSP and NIC Conditioning

The aim of this experiment was to verify whether the training of male *B. germanica* to either TSP-extract- or nicotine-affected subsequent behavior. This experiment was carried out in three distinct phases and is summarized in Table 2.

#### Phase 1—Training Phase

In phase 1, 2 groups of 12 males were randomly selected 7 d after the last molt and placed in separate 1-liter airtight containers equipped with a drilled lid for ventilation. Each container was provided with a water dispenser and a cardboard strip as harborage. In the first container, males were offered TSP-extract-treated food in solid form (0.5 g) inside a 1.5-ml Eppendorf tube. In the second container, males were allowed to feed on nicotine-treated food (NIC) administered in solid form (0.5 g). The amount of food consumed in phase one was recorded. Water evaporation was also considered.

#### Phase 2—Stabilization Phase

After 7 d, each of the two groups from phase 1 was divided into three subgroups (Table 2): the first subgroup was starved for 72 h, the second subgroup continued to feed on the TSP-extract- or nicotine-treated food, while the third subgroup was reestablished on the control food diet. Similar to the first phase, all feeds were administered in solid form. Water evaporation was considered.

#### Phase 3—Choice Phase

Similar to the choice phase of experiment 1, in phase 3, male cockroaches were placed individually into round 1-liter airtight containers and allowed to make a choice between treated and control liquid food administered by 25-µl glass capillaries.

Males treated in the first phase with TSP-extract-treated food, and put under the three different food regimes in the second phase as described above, could only choose between control (BLA) and TSP-extract-treated food (TSP), whereas the individuals fed with nicotine-treated food in the first phase, and separated into three different feeding regimes during the second phase, could only choose between control (BLA) and nicotine-treated food (NIC; Table 2). The amount of food consumed by single males in each container was recorded daily according to the method described above. The experiment was repeated four times.

### Experiment 3—Olfactometry Bioassays

Olfactometry bioassays were conducted to verify the odor-mediated preference of male *B. germanica* for the TSP extract or control food. Experiments comprised three distinct phases.

#### Phase 1

Ten males were placed in a 1-liter airtight container fitted with a lid containing drilled holes for internal ventilation, provided with a water dispenser and a cardboard strip as harborage. Food was offered in solid form inside a 1.5-ml Eppendorf tube continuously for 5 d. Three treatment groups were prepared: a control group (BLA) where individuals were fed on control food, a TSP group where the males fed on TSP-extract-treated food, and a starved group where no food was provided.

#### Phase 2

All the treatment groups from phase 1 were starved for 48 h.

#### Phase 3

Individual males were allowed to make a choice between two olfactory stimuli presented in a Y-tube olfactometer, which was assembled such that the insects were unable to come into contact with the odor sources kept in airtight glass vials placed upstream to the Y-shaped arena. The olfactometer comprised a one-piece Y-shaped glass tube with an internal diameter of 2 cm. The two arms and the stem of the Y-tube were equal in length (22 cm). A 5-cm-long glass tube expansion located on the stem of the Y-tube served as the entry point for an individual cockroach and functioned as an acclimatizing chamber. Male cockroaches were anesthetized by keeping at −18°C for 2 min and then placed individually in the acclimatizing chamber. Air entering the system was purified by means of a carbon filter and then humidified by passing through distilled water. The flow rate was set at 150 ml/min. Bioassays were carried out in the dark, under a dim red light to avoid light interference. Variables recorded were 1) the time spent by each male cockroach in either arm of the Y-tube olfactometer and 2) the first choice and the total number of entries per minute. Each experiment lasted 5 min, and experiments were replicated four times. After testing ten individuals, the connections of the odor sources to the olfactometer arms were exchanged to remove any asymmetrical bias in the set up, and the odor sources were replenished with fresh material. At the end of each day, the Y-tube and glass vials were thoroughly washed with soap, rinsed with ethanol (90%) and dried overnight in an oven at 200°C.

### Statistical Analysis

Bioassay data were analyzed using Student t-test and one-way analysis of variance (ANOVA) using SPSS for Windows.

## Results and Discussion

### Experiment 1—Nicotine and TSP Innate Preference

The consumption of artificial food by male *B. germanica* was recorded in the first phase of experiment 1. Nonstarved insects consumed on average 4.15 ± 1.01 mg of food per day per individual. In the second phase, there was no statistically significant difference in the total amount of food consumed from capillaries by cockroaches that were either starved or were allowed to feed in the first phase (*t* = 1.88; *P* = 0.07; df = 61), suggesting that the regime of starvation did not alter food intake in the second phase (Table 1).

**Fig. 1.**
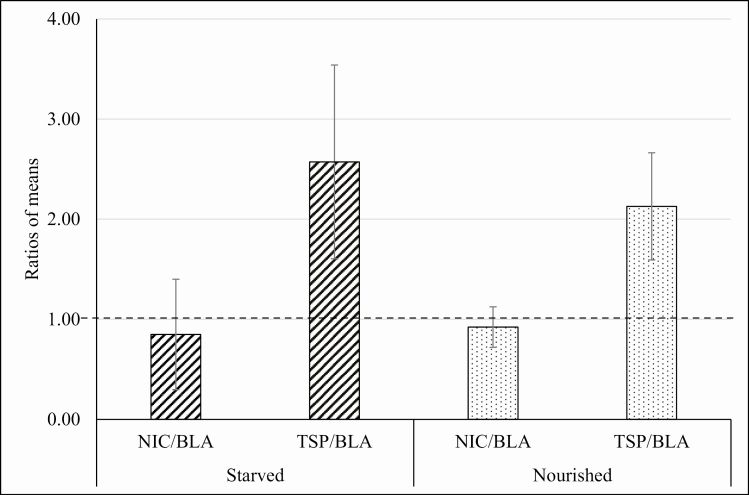
Experiment 1. Average individual consumption of experimental food during the choice phase. The values are obtained by a ratio between the average consumption of TSP- or NIC-treated food and blank control food (BLA) in the choice phase. Therefore, the value 1 represents an equal consumption of food (dotted bold line). Ratio values over 1 show preference for treated food over the control, whereas values between 0 and 1 reveal a preference for control food. SD are presented as error bars. The total consumption of food of starved and nourished individuals was not significantly different at *P* = 0.05. Preference for TSP-treated food over the control food was highly significant for both the starved and the nourished cockroaches. No significant difference was detected between the average consumption of blank control food and NIC-treated food at *P* = 0.05 for both the starved and the nourished individuals.

In Fig. 1, data regarding the choice assays in experiment 1 were presented as ratios between the average consumption of either TSP-extract- or NIC-treated food, and control food, when offered as a choice from microcapillaries. Therefore, a value of 1 represented an equal consumption of food from the two capillaries; a value greater than 1 suggested a preference for treated food over control food; a value between 0 and 1 suggested a preference for control food. TSP-extract-treated food was consumed more than control food for both starved (*t* = 5.62; *P* < 0.01; df = 30) and fed (*t* = 3.78; *P* < 0.01; df = 30) male cockroaches (Fig. 1). The ratio between the mean consumption of TSP-extract-treated food and control food was largely greater than 1 for both the starved and fed group, indicating a highly significant preference for the TSP-extract-treated food over the control food. By contrast, there was no statistically significant difference in consumption of NIC-treated food and control food (starved *t* = 1.04; *P* = 0.31; df = 30; nourished *t* = 0.96; *P* = 0.34; df = 30). The ratio of NIC/BLA was close to 1 for both starved and fed cockroaches (Fig. 1), confirming that there was no significant preference for the NIC-treated food over the control food.

### Experiment 2—TSP and NIC Conditioning

Experiment 2 was aimed to evaluate the effects of training on the choices of the insects and to obtain clues about the onset of a status of addiction. The experiment was therefore divided into three separate phases: a first training phase, a second stabilization phase and a third choice phase. In the first phase, no significant differences were recorded between the consumption of TSP- and NIC-treated food (Table 3). Male cockroaches fed with TSP- or NIC-treated food consumed the same amount of food. This amount was consistently similar to the intake of control food observed in the phase 1 of experiment 1. TSP-extract-treated insects consumed on average 4.07 ± 0.57 mg of food per day per individual and NIC-treated cockroaches consumed on average 4.63 ± 0.52 mg of food per day per individual.

**Table 3.** Experiment 2. Mean ratios of food consumption in phase 1 (left) and in phase 2 (right) given in mg per individual

Phase 1 (7 d)			Phase 2 (3 d)		
Treatment group	Mean	SD	Treatment group	Mean	SD
Nicotine (NIC)	32.42	3.66	Starved	—	—
			Control	31.04	4.27
			NIC	33.96	8.59
Tobacco smoke particulate matter extract (TSP)	28.46	3.96	Starved	—	—
			Control	30.00	4.08
			TSP	34.17	3.91

The SD are indicated on the right of the means. The Student *t*-test shows that the difference between the quantity of food consumed in the first phase between NIC- and TSP-treated food is not significantly different (*t* = 1.4683; *P* = 0.1924; df = 6). As regards the second phase, the difference between control food and NIC-treated food is not significantly different (*t* = 0.6081; *P* = 0.5654; df = 6). Likewise, also the difference in the intake of control food and TSP-treated food is not significantly different (*t* = 1.4744; *P* = 0.1908; df = 6). In the second phase, the total food ingestion (control + NIC) of the cockroaches treated with NIC-treated food in the first phase is not significantly different from the total amount of food (control + TSP) consumed by the individuals treated with TSP-treated food in the first phase (*t* = 1.1515; *P* = 0. 8817; df = 14).

During the second phase, male cockroaches treated with TSP-extract-treated food were divided into three groups: the first group was starved for 3 d, the second group received control food, and the third group continued to feed on TSP-extract-treated food. Similarly, insects that fed with NIC-treated food during the first phase were divided into three groups, that is, a starved group, a group in which control food was provided, and a third group that continued to feed on the NIC-treated food. When the total intakes of food in the first and second phases were compared, the average individual quantities consumed by the cockroaches were not statistically different. However, considering that the first phase lasts for 7 d while the duration of the second phase is 3 d, a significant increase of the daily individual consumption of food was observed. Individuals of the control group in the second phase, trained with NIC in the first phase, consumed an average of 10.35 ± 1.42 mg per day, while males that continued to feed on NIC consumed, in the second phase, an average of 11.32 ± 2.86 mg per day per individual. Similarly, males that were fed with TSP in the first phase and were fed with control food in the second phase, consumed an average of 10.00 ± 1.36 mg per individual per day. Males that continued to feed on the TSP-treated food consumed 11.39 ± 1.31 mg per individual per day.

**Fig. 2.**
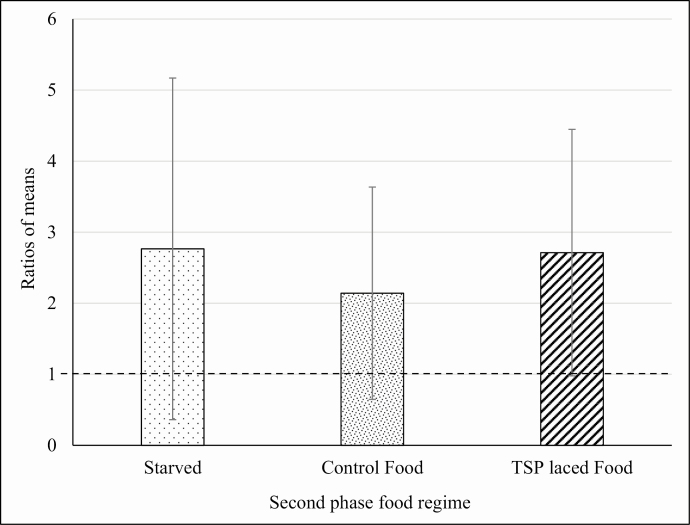
Experiment 1. Average ratios of consumption of experimental food during the choice phase for the insects trained on TSP. The values are obtained by a ratio between the average consumption of TSP-treated food and control food in the choice phase. Therefore, the value 1 represents an equal consumption of the two types of food (dotted bold line). Ratio values over 1 show preference for TSP food over the control. SD are presented as error bars. The total consumption of food in the three treatment groups was not significantly different at *P* = 0.05. Preference for TSP-traced food over the control food was significant for the starved group (*t* = 2.9611; *P* = 0.0059; df = 30), the control group (*t* = 2.4073; *P* = 0.0224; df = 30) and the TSP group (*t* = 3.807; *P* = 0.0006; df = 30).

**Fig. 3.**
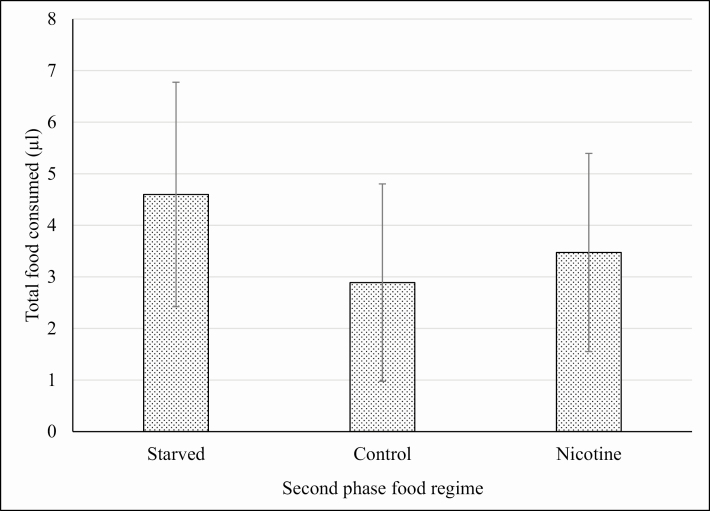
Experiment 2. Average individual consumption of experimental food during the choice phase for the insects trained on NIC-treated food. SD are presented as error bars. The total consumption of food in the three treatment groups was significantly different at *P* = 0.05. Total intake of food of the Starved group was significantly higher than the other groups (*F* = 3.9493; *P* = 0.0265; df = 47).

**Fig. 4.**
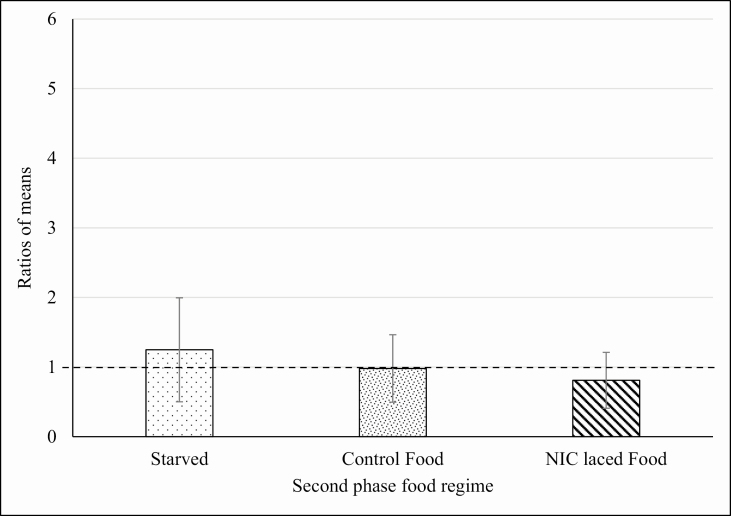
Experiment 2. Average ratios of consumption of experimental food during the choice phase for the insects trained on NIC-treated food. The values are obtained by a ratio between the average consumption of NIC-treated food and control food in the choice phase. Therefore, the value 1 represents an equal consumption of the two types of food (dotted bold line). Ratio values over 1 show preference for NIC-treated food over the control, whereas values between 0 and 1 reveal a preference for control food. SD are presented as error bars. Preference for NIC-treated food over the control food was not statistically significant for any group of treatment.

These data suggest that there was a general increase in the appetite of the insects in all treatment groups (Table 3), probably to a phagostimulant effect induced by nicotine. Moreover, in the second phase, the statistical analysis showed that there was no significant difference in food intake between the individuals that continued to feed on the TSP-extract- and NIC-treated foods, and those that fed on the control food. Some of the insects trained on the NIC- and TSP-extract-treated food were deprived of food during the stabilization phase to check for eventual relapse-like effects induced by nicotine or TSP extract that could be observed in the third phase of choice. For the third phase, within the TSP-extract-trained group, all the insects were offered a choice between TSP-extract-treated food and control food. ANOVA showed that there was no significant difference in the total amount of food consumed by the insects in the third phase that were kept in the different food regimes during the second phase (starvation, feeding on control and TSP-extract-treated food; *F* = 0.5467; *P* = 0.5826; df = 47). This analysis suggests that, in the experimental conditions used, feeding regime does not affect the feeding behavior of the insects trained on TSP-extract-treated food. However, in the choice test, a highly significant preference was observed for the glass capillaries containing the TSP-extract-treated food compared with glass capillaries that contained control food, despite the different food regimes during the previous phase (starved: *t* = 2.9611; *P* = 0.0059; df = 30; Control food: *t* = 2.4073; *P* = 0.0224; df = 30; TSP-treated food: *t* = 3.807; *P* = 0.0006; df = 30; Fig. 2). The preference was not due to the spatial arrangement of the glass capillaries inside the container because, in preliminary tests where the glass capillaries contained the same diet, no significant differences were found in the consumption of food from the two sources. Similar to that described for TSP-extract-trained males, NIC-trained males underwent either starvation or feeding with control food or NIC-treated food in the second phase. In third phase, all these insects were offered a choice between NIC-treated food and control food. In contrast to that observed for TSP-extract-trained males, ANOVA showed that the total amount of food consumed by starved insects in the choice phase was significantly higher (*F* = 3.9493; *P* = 0.0265; df = 47) than other groups of treatments in the second phase (Fig. 3). There was no significant difference in the choice between NIC-treated food over the control food in any of the three subgroups of the second phase (Fig. 4). This lack of preference for control or NIC-treated food suggests that the increase in the total consumption of food observed for the starved group of males (Fig. 3) is not due to the onset of an addiction-like effect, but due to phagostimulation induced by nicotine, consistent with the data from the second phase discussed above. By contrast, consumption of food by TSP-trained males starved in the phase 2 did not show any significant difference when compared with other groups. It is still possible to hypothesize phagostimulation induced by nicotine, as both NIC- and TSP-treated foods contained exactly the same amount of nicotine that could act as a phagostimulant at the concentration used but result in marked preference of TSP-treated food over the control. El-Heneidy et al. (1988) found that at moderate nicotine concentrations, fall armyworm larvae, *Spodoptera frugiperda* Smith, 1797 (Lepidoptera: Noctuidae), gained weight due to direct phagostimulation. Moreover, it was recently reported that nicotine is repellent at high concentrations, while enhancing the learning performance of bumblebees, *Bombus terrestris audax* (Harris, 1776) (Hymenoptera: Apidae) during pollination, thereby suggesting that the volatile nature of the free base alkaloid may contribute to the activation of gustatory sensilla (Kessler et al. 2015, Baracchi et al. 2017). Other authors suggest that the effects of nicotine are dependent on the context in which the alkaloid is applied; context dependency of alkaloid effects is not limited to the environment in which alkaloids are presented to the insect (i.e., natural or artificial substrates) but can also be influenced by other factors like prior feeding experience (Shields et al. 2006). The choices made by male German cockroaches in experiment 2 following setting of a training and the stabilization phase was in line with these previous studies; however, both phases had no effects on the subsequent choice of the male cockroaches. Taken together, our data clearly show that male cockroaches preferred TSP-extract-treated food compared with the control food, while no difference in preference was detectable when NIC-treated food was offered versus the BLA food. Moreover, males trained in the first phase with TSP-extract-treated food always preferred the TSP-extract-treated food over the control food whatever their diet in the second phase. The insects continued to express a significant preference for the TSP-extract-treated food when a control food diet was reestablished in the stabilization phase. Insects trained on TSP extract food preferred this food over the control food when they were starved or continued to feed on TSP-extract-treated food or even when they were offered the choice to feed on control food (Fig. 2). It was also found that different regimes in the stabilization phase did not interfere with the preference for TSP extract food in the choice phase. In fact, statistical analysis showed no significant differences among the food consumption of the TSP-trained males subjected to different food regimes in the second phase. This suggests that the TSP-extract-treated food was always preferred over the control no matter what was offered in the training and in the stabilization phase.

For nicotine-trained male cockroaches, no significant preference for the nicotine-treated food compared with the control food was observed (Fig. 4). Since the ratio of consumption from the two glass capillaries for control and nicotine-treated food was close to 1 (Fig. 4), it can be assumed that male cockroaches do not express any preference in the choice between these two types of food (Fig. 4). This observation was the same for all the treatment groups in the stabilization phase and was confirmed by the statistical analysis that showed no statistical differences among subgroups of the NIC training cluster. This observation is consistent with the data coming from the experiment 1 where no preference was observed between control and nicotine-added food and suggests that, as far as male German cockroaches are concerned, nicotine is not responsible for the observed preference for TSP-extract-treated food.

### Olfactometer Assays

**Fig. 5.**
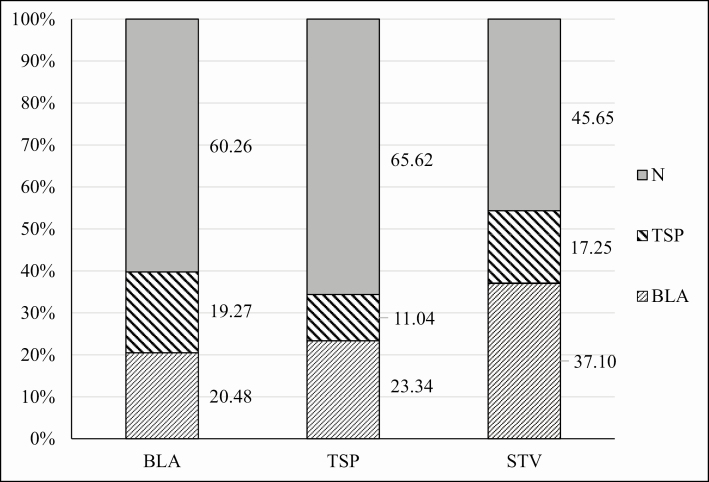
Experiment 3, olfactometer bioassays. Time spent in each arm of the olfactometer by males trained on TSP or BLA food or deprived of food. *N* indicates the mixing arm of the olfactometer. Values are given as a percentage of the total time of the test. Differences in the time spent in the two treatment arms of the olfactometer were not statistically significant.

**Fig. 6.**
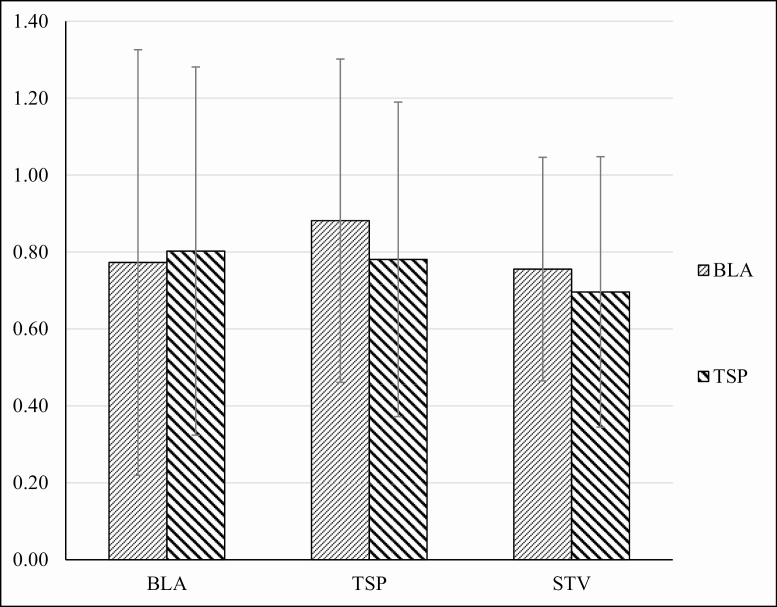
Experiment 3, olfactometer test. Number of entries per minute in each of the treatment arms of the olfactometer. SD for each group are presented as error bars. Differences were not statistically significant.

The results of the choice tests, which showed that *B. germanica* had a strong preference for the TSP-extract-treated food over control food, could be due to olfaction, a gustatory preference or the onset of an addicted status. To determine whether olfaction had a role in expression of the preference, olfactometer assays were carried out with male cockroaches and TSP-extract-treated food as the odor source. As cockroaches showed no significant interest for the nicotine-treated food in choice tests, this food source was not tested in the olfactometer. Although the cockroaches were fast-moving and could visit all zones of an olfactometer several times during the experimental period, data collected revealed that there was no significant olfactory preference for the odor of TSP-extract-treated food over the odor of control food and that males spent most of their time in the stem of the Y-tube where they were exposed to both odors (Fig. 5). Males deprived of food in the first phase spent less time in the stem than the other treatment groups. This behavior was probably due to the higher need to feed regardless of the source. In particular, starved insects spent more time in arm containing the odor of the control food than in the arm containing the odor of the TSP-extract-treated food, suggesting that the latter was not attractive as a food source when the insects made the choice only on an olfactory basis (Fig. 5). Comparing the time spent in the two treatment arms without considering the stem, statistical analysis did not indicate a significant preference between the TSP-extract-treated food and control food odors. The number of visits of the two treated arms was also not significantly different (Fig. 6).

It has been reported that the effects of alkaloids such as nicotine, originally considered to be potent feeding deterrents, are highly variable, depending on the substrate on which they were presented (Shields et al. 2006). Gustatory, olfactory, tactile, somatosensory, habituation, or toxicity-based mechanisms may be playing roles in determining the effect of nicotine in deterring feeding (Glendinning 1996). Shields et al. (2006) hypothesized that gustatory information is pivotal in determining the effect of alkaloids on feeding of Gypsy moths, *Lymantria dispar* (Linnaeus) (Lepidoptera: Erebidae). Our data indicate that *B. germanica* has a strong innate preference for TSP extract and that this preference, observed in choice tests, is due to a gustatory choice rather than through an olfactory-mediated mechanism. This hypothesis is reinforced by an increase in the total amount of food consumed in the second and third phases of the choice tests by cockroaches exposed to nicotine and TSP extract in the first phase. It is possible that the increased feeding response could also be due to the onset of an addicted-like status induced by TSP, but further experiments are required to test this hypothesis of addiction. As TSP-extract-treated cockroaches showed a preference for TSP-treated food over control food in choice tests, phagostimulation cannot account for this preference as insects would be expected to feed equally from both sources in choice tests. On the other hand, phagostimulation could account for the results of choice tests with nicotine-treated cockroaches, as there was no significant preference for nicotine-treated food or control food.

As mentioned above, data concerning the TSP-extract-treated food could also support an addiction hypothesis. Cockroaches fed on TSP-extract-treated food showed an increase of food consumption over time, which is a criteria of the addiction paradigm (Kaun et al. 2012), and also exhibited a preference independent from any olfactory input, as demonstrated by the olfactometer assays. Also, it has been reported that nicotine is weakly reinforcing and does not account alone for the addictive effects of tobacco in human subjects (Ambrose et al. 2007, Lewis et al. 2007). Moreover, among mammals, components of the tobacco smoke, other than nicotine, play a key role in the onset of addiction (van Amsterdam et al. 2006, Ambrose et al. 2007, Lewis et al. 2007, Brennan et al. 2015). Taken together, our data suggest phagostimulation effects elicited by both nicotine and TSP extract, but could also suggest the onset of addiction. Further experiments aimed to assess withdrawal, relapse effects and overcoming of aversive stimuli are indeed necessary to test the hypothesis of addiction. Given the difference in the response of male cockroaches following the application of TSP extract compared with nicotine, which is also present in the TSP extract, TSP extract may contain bioactive compounds that either are effective themselves or enhance the properties of nicotine. In this context, it is necessary to run further experiments to identify the compounds responsible for the observed effects and what is the relationship between these molecules and the nicotine.

### Conclusions

Using a combination of feeding bioassays and olfactometer assays in this study, we have demonstrated that male German cockroaches, *B. germanica*, prefer TSP-treated food compared with nicotine-treated food or control food. In choice tests, TSP-treated food was significantly preferred over control food, which in turn did not elicit a different feeding response compared with nicotine-treated food. Nicotine, which is commonly recognized as the most important addictive substance from tobacco, did not induce addictive effect on cockroaches. Both TSP and nicotine-treated food appeared to possess phagostimulant properties as the consumption of food increased during the experimental period. This result is partially in contrast with the literature, which identifies nicotine as a feeding deterrent. However, variability of the response to nicotine and other alkaloids in relation to the context in which they are applied needs to be taken into consideration. In addition, the phagostimulatory effect does not explain alone the marked preference for TSP observed in the choice tests of the experiments that may be due to a gustatory preference or to the onset of an addiction status.

Olfactometry experiments suggest that the preference for TSP is not connected with an olfactory stimulation and supports the hypothesis that tobacco-related products can elicit either an addiction-like status or a gustatory response because cockroach males need to consume the treated food to express a preference. The results of this study suggest that TSP extract contains compounds that either are bioactive in their own right or enhance the properties of nicotine. In this context, it is crucial to identify the compounds responsible for the observed effects and what is the relationship between these molecules and nicotine. Finally, further experiments are planned to determine whether TSP induces addiction by promoting the release of neurotransmitters and if there is a concomitant inhibition of monoamine oxidases, as reported in human subjects.
